# Ionomic Screening of BRRI dhan84 Mutagenized Population Identifies Candidate Genes Underlying High Arsenic and Low Zinc/Cadmium Accumulation

**DOI:** 10.1111/ppl.70980

**Published:** 2026-06-25

**Authors:** Shihab Uddin, Md. Rafiqul Islam, Mirza Mofazzal Islam, Md. Abdul Kader, Toru Fujiwara, Takehiro Kamiya

**Affiliations:** ^1^ Graduate School of Agricultural and Life Sciences The University of Tokyo Tokyo Japan; ^2^ Department of Soil Science Bangladesh Agricultural University Mymensingh Bangladesh; ^3^ Plant Breeding Division Bangladesh Institute of Nuclear Agriculture Mymensingh Bangladesh; ^4^ Plant Breeding Division Bangladesh Rice Research Institute Gazipur Bangladesh

## Abstract

Mutagenesis combined with ionomic profiling offers a powerful approach to explore the genetic basis of essential and toxic element accumulation in rice grain and to generate materials for marker‐assisted breeding. However, large‐scale ionomic screening has not previously been conducted in Bangladeshi rice germplasm. To isolate mutants with altered grain concentrations of iron, zinc (Zn), arsenic (As), and cadmium (Cd), we mutagenized BRRI dhan84 using ethyl methanesulfonate (EMS; 0.5% and 1%) and gamma rays (250, 300, 350, and 400 Gy). A total of 8503 M_2_ plants were screened for brown rice ionome using inductively coupled plasma mass spectrometry (ICP‐MS). Candidate mutants were selected based on robust *Z*‐scores (|*Z*‐scores| > 2) of the elements for individual plants, resulting in the isolation of 38 mutants. To validate the screening process, two mutants were characterized in detail: high grain As (*osabcc1‐3*) and low grain Zn and Cd (*oshma2‐4*). Whole genome sequencing of the mutants and the correlation between phenotype and genotype in the F_2_ population indicated that *OsABCC1* and *OsHMA2* are likely the causal genes. The *osabcc1‐3* mutant carries a splice‐site mutation at the exon‐intron junction of *OsABCC1*, whereas *oshma2‐4* harbors a 3.3 kb insertion in *OsHMA2*. Notably, *oshma2‐4* exhibited similar growth and yield to BRRI dhan84 in a paddy field, suggesting its potential for breeding low‐Cd rice. Together, these results demonstrate the effectiveness of ionomic screening in Bangladeshi rice for uncovering allelic variation controlling metal homeostasis and provide valuable genetic resources and physiological insights relevant to rice improvement.

## 1 Introduction

The ionome, defined as the complete profile of mineral nutrients and trace elements within an organism, represents the inorganic composition of biological systems (Salt et al. 2008). Element accumulation in plants is a complex multigenic process influenced by uptake from soil, root‐to‐shoot translocation, and sequestration in specific tissues, and variation in a single gene often affects the accumulation of multiple elements (Singh et al. 2020; Saito et al. 2026). This is particularly important in rice ( *Oryza sativa* ), a staple food crop, because grain concentrations of essential micronutrients such as iron (Fe) and zinc (Zn) are critical for human nutrition, whereas toxic elements such as arsenic (As) and cadmium (Cd) pose major food safety risks. Ionomic profiling enables the simultaneous quantification of essential and toxic elements and provides an efficient phenotyping approach for identifying genetic variation controlling grain mineral composition. When combined with genetic and genomic tools, ionomic screening has contributed to the identification of key loci and genes regulating elemental homeostasis and has become an effective strategy for developing breeding materials with improved grain nutritional quality and reduced toxic element accumulation (Ishikawa et al. 2012; Kandwal et al. 2022; Yan et al. 2023).

Chemical and physical mutagenesis complements ionomic profiling by generating novel allelic variants that exhibit altered element accumulation. Forward genetic screening of mutagenized populations, combined with ionomic analysis, has proven effective for identifying genes involved in elemental homeostasis. Early studies in *Arabidopsis thaliana* established the value of this approach by demonstrating that ionomic phenotypes can be used to discover genes controlling mineral homeostasis and root barrier function (Huang and Salt 2016). These pioneering efforts provided a conceptual framework for applying ionomic screening to crop improvement through the identification of genes underlying multi‐element phenotypes.

In rice, forward‐genetic approaches coupled with ionomic screening have enabled the identification of both known and novel genes regulating the accumulation of toxic and essential elements in grain. For example, analysis of mutants with elevated grain As led to the identification of *OsABCC1* and *OsPCS1*, which contribute to As detoxification and sequestration (Hayashi et al. 2017). Similarly, mutant‐based screening identified new alleles of *OsNramp5* and *OsHMA3*, which are major determinants of Cd uptake and vacuolar sequestration, respectively (Tanaka et al. 2016). In addition, genes controlling molybdenum (Mo) distribution and accumulation, such as *OsMOT1;2* and *OsDISMO1*, have been characterized through mutant‐based approaches (Ishikawa et al. 2021; Kandwal et al. 2025). Collectively, these studies highlight the effectiveness of ionomic screening as a forward genetic strategy for identifying functional alleles relevant to grain quality improvement.

Previous studies have further shown that mutagenesis can expand genetic diversity and provide practical breeding materials for improving grain elemental composition in *japonica* rice. For instance, Ishikawa et al. (2012) developed a carbon‐ion‐beam‐irradiated mutant population of the japonica cultivar Koshihikari and isolated mutants with reduced accumulation of toxic metals such as Cd, cesium, and As. The *lcd‐kmt2* mutant, one novel allele of *OsNramp5* encoding a Mn transporter, accumulated less Cd in grain compared with the wild type (WT) (Ishikawa et al. 2012). The *lcs1* mutant, defective in *OsSOS2*, accumulated less cesium in grain, while the *las‐3* mutant, an allele of *ADH2*, showed reduced As accumulation (Ishikawa et al. 2017; Hayashi et al. 2021). Similarly, Tanaka et al. (2016) generated an EMS‐mutagenized population of the japonica cultivar Hitomebore and isolated some mutants with beneficial ionomic phenotypes. Among these, the *1281_m* mutant, another novel allele of *OsNramp5*, accumulated significantly lower levels of Cd, whereas the *1095_k* mutant, an allele of *OsVIT2*, showed significantly increased Fe and Zn concentrations compared to the WT (Tanaka et al. 2016; Kandwal et al. 2022). Recently, Saito et al. (2026) established a non‐transgenic Fe‐biofortified rice line from the Taichung‐65 (T65) background through novel mutation in the *HRZ1* gene. Notably, these mutants exhibited growth and yield comparable to the WT under paddy field conditions and were proposed as promising breeding materials. Because these mutants are non‐transgenic, they are readily acceptable in global markets (Grover et al. 2020). Moreover, identification of the causal mutations has enabled their effective use in marker‐assisted breeding for improving rice quality. Despite these advances in japonica rice, comparable large‐scale mutagenized populations and well‐characterized ionomic mutants remain largely unavailable in *indica* rice.

In Bangladesh, micronutrient malnutrition, particularly Fe and Zn deficiency, and exposure to toxic elements such as As and Cd through rice consumption remain major public health concerns. Given the high per capita consumption of rice (FAO 2022), improving grain mineral composition is a long‐term goal for enhancing nutritional security and food safety. While Zn‐ and Fe‐enriched rice varieties have been developed through conventional breeding, their adoption remains limited (Nutrition Connect 2019), and cultivars specifically selected for low As or low Cd accumulation are not yet available in Bangladeshi rice germplasm. Moreover, systematic ionomic screening and marker‐assisted breeding approaches have been minimally applied to Bangladeshi rice cultivars, and mutagenized populations remain largely unexplored as genetic resources for studying elemental accumulation and breeding purposes.

To establish novel non‐transgenic breeding materials with improved grain element composition, we applied large‐scale ionomic screening to a mutagenized population of the Bangladeshi *indica* rice cultivar BRRI dhan84. The main objectives of the study were to (1) identify and select mutant lines showing beneficial grain phenotypes, including high Fe/Zn and low As/Cd accumulation, through ionomic screening, and (2) demonstrate the utility of this population by characterizing representative mutants and identifying candidate causal genes underlying their phenotypes.

## 2 Materials and Methods

### 2.1 Planting Materials and Growing the Screening Population

BRRI dhan84, a Zn‐enriched Bangladeshi rice cultivar developed by the Bangladesh Rice Research Institute (BRRI) (Kader et al. 2020), was used for mutagenesis. The seeds of BRRI dhan84 were mutagenized with different levels of ethyl methanesulfonate (EMS; 0.5% and 1%) and gamma rays (250, 300, 350, and 400 Gy). The M_1_ seeds were grown in the paddy field of Bangladesh Agricultural University (BAU) (24°42′57.6″ N 90°25′32.5″ E) during the boro (dry) season (December 2020–May 2021), and three panicles from each plant were harvested. Using the three panicles (M_2_), two populations were screened: population A and population B. For population A, one M_2_ seed from a single panicle was germinated and grown in a farmer's field in Faridpur (23°35′12.0″ N 89°47′50.4″ E), an As‐contaminated area, during the boro season (December 2021–May 2022), and five panicles were harvested. For population B, one M_2_ seed from a mixture of another two panicles was germinated and grown in the BAU field (24°42′55.5″ N 90°25′30.2″ E) during the Aman (monsoon) season of the same year (July–November 2022). This approach was used because previous studies have shown that M_2_ plants derived from different tillers of a chemically mutagenized rice M_1_ plant carry independent sets of mutations (Yamazaki et al. 2023). Five mature panicles were harvested from each M_2_ plant. One panicle was selected, air‐dried, and dehusked to obtain brown rice. Five brown rice grains were used for ionome screening by ICP‐MS, and mutants were selected. The selected M_3_ plants from populations A and B were grown in the BAU field during the boro season (December 2022–May 2023) and the boro season (December 2023–May 2024), respectively. The selected M_4_ plants from population A were grown in the BAU field (24°42′56.7″N 90°25′32.2″ E) during the boro season (December 2023–May 2024).

### 2.2 Preparation of F_2_ Populations for Segregation Analysis

For segregation analysis, the selected mutants were crossed with either WT (BRRI dhan84) or an EMS‐mutagenized line (*1%EMS_L13*). While *1%EMS_L13* has similar ionome phenotypes to the WT, it is expected to carry thousands of mutations by EMS (Figure S1). The mutant was crossed with *1%EMS_L13* when it was a gamma‐ray mutagenized mutant; since gamma‐ray mutagenesis typically induces fewer mutations, crossing with a highly mutated line like *1%EMS_L13* was necessary to generate sufficient polymorphisms for designing DNA markers and identifying causal genes. Two independent heterozygous F_1_ plants from each cross were grown to obtain the F_2_ seeds.

### 2.3 Hydroponic Cultivation Method

Seeds were surface‐sterilized with 50% sodium hypochlorite solution containing 2–3 drops of detergent for 15 min, rinsed thoroughly with ultrapure water, and soaked in ultrapure water for germination at 28°C under a 16/8 h light/dark cycle for 3 days in a growth chamber. Then uniformly grown seedlings were selected and transferred to Kimura B solution (Baba and Takahashi 1956) with or without As or Cd and grown for an additional 12 days. Initially, the solution was renewed on days 3 and 6 because the seedlings were still small; thereafter, it was renewed every alternate day until harvest to maintain stable nutrient concentrations. Two‐week‐old plants were harvested, and shoots and roots were oven‐dried for the determination of element concentrations.

For the sensitivity experiments, plants were cultivated in Kimura B solution (pH 5.8) with varying levels of As, Cd, and Zn, and growth parameters were recorded at 2 weeks for As and 3 weeks for Cd and Zn, respectively. The nutrient solution was renewed as described before. The nutrient composition of Kimura B solution has been provided in Table S1. The growth parameters were recorded at different plant ages depending on the expected toxicity dynamics and physiological response to each element. Specifically, because As toxicity symptoms appeared more rapidly, plants were evaluated after 2 weeks of exposure. In contrast, Cd required longer exposure periods to exhibit clear and measurable phenotypic differences and was therefore assessed at 3 weeks. These specific time points were selected based on preliminary observations to capture the maximum genotype‐dependent variation without causing severe mortality that could mask these differences.

### 2.4 Determination of Element Concentrations

The brown rice, shoot, and root samples were dried at 70°C for 72 h in an oven. The dried samples were then digested with 4 mL of HNO_3_, followed by 2 mL of H_2_O_2_, at 100°C until the solution was completely evaporated. After this digestion, the resulting precipitate was dissolved with 2 mL of 0.08 N HNO_3_. After dilution, the element concentrations were determined by inductively coupled plasma mass spectrometry (ICP‐MS) (Agilent 7800; Agilent Technologies), with indium serving as an internal standard. To ensure a reliable quantification, we confirmed that the sample concentrations were higher than the concentration of the lowest calibration standard. Furthermore, to ensure the accuracy and reliability of our ICP‐MS analysis, we validated our analytical method using a standard reference material (SRM 1573a, Tomato Leaves; NIST). We performed five technical replicates, and the recovery rates for the target elements were shown in Table S2.

### 2.5 Calculation of Robust *Z*‐Score

For screening purposes, the grain element concentrations were used to calculate the robust *Z*‐score for each element using the following equation:

$$\text{Robust}\;Z\;-\;\text{score}=\frac{X_{i}-X_{m}}{\text{NIQR}}$$

where *X*_*i*_ = log_10_ value of the sample; *X*_*m*_ = median log_10_ value of the samples analyzed by ICP‐MS in 1 day (200 ~ 300 samples); NIQR = normalized interquartile range.

### 2.6 Whole Genome Resequencing by Next‐Generation Sequencing

Total DNA was extracted from the shoots of 2‐week‐old mutant and WT (BRRI dhan84) seedlings using the DNeasy Plant Mini Kit (QIAGEN). Sequencing libraries were prepared and subjected to paired‐end sequencing (150 bp) using the DNBSEQ platform (BGI). Raw sequencing reads (FASTQ formats) were quality‐checked by fastp (Chen 2023) and mapped to the rice reference genome ( *Oryza sativa* ssp. *japonica* cv. Nipponbare, IRGSP‐1.0: https://rapdb.dna.affrc.go.jp/download/irgsp1.html) using Bowtie2 (Langmead and Salzberg 2012). The resulting alignment files were used for variant detection using the HaplotypeCaller function of GATK4 (DePristo et al. 2011).

### 2.7 Direct Sequencing Analysis

To determine the genomic DNA sequence of *oshma2‐4*, DNA was extracted from the mutant using Edwards solution (Edwards et al. 1991; Kasajima et al. 2004). To confirm the predicted mutation site (junction between chromosomes 6 and 10), the *OsHMA2* genomic region spanning the junction was amplified by PCR using GoTaq DNA polymerase (Promega) with the primer pair *OsHMA2*_F and *OsHMA2*_R2 (Table S2). The PCR product was subjected to Sanger sequencing using the *OsHMA2*_Junc1_Seq primer (Table S3).

To determine the cDNA sequence of the *oshma2‐4* mutant, total RNA was extracted from roots of the mutant seedlings, and first‐strand complementary DNA (cDNA) was synthesized using SuperScript IV (Thermo Fisher Scientific). The partial sequence of *OsHMA2* (*Os06g0700700*) was amplified from both genomic DNA and cDNA by PCR using GoTaq DNA polymerase (Promega) and the *OsHMA2*_F and *OsHMA2*_R1 primers (Table S3). To confirm the mutation site (junctions of chromosomes 6 and 10), the partial sequence of *OsHMA2*, including the mutation site, was amplified using GoTaq DNA polymerase (Promega) and the *OsHMA2*_F and *OsHMA2*_R2 primers (Table S3). The sequence was then determined by Sanger sequencing using the *OsHMA2*_Junc1_Seq primer (Table S3). The partial sequence of *OsHMA2* from exon 2 to exon 9 was amplified using cDNA as a template by PCR with PrimeSTAR Max DNA polymerase (TaKaRa) and *OsHMA2*_Exon2_F and *OsHMA2*_Exon9_R primers (Table S3).

For the determination of the *osabcc1‐3* cDNA sequence, roots of two‐week‐old WT and *osabcc1‐3* plants were frozen in liquid N_2_ and stored at −80°C until RNA extraction. Total RNA was extracted using the NucleoSpin RNA Plant kit (TaKaRa), and first‐strand cDNA was synthesized using PrimeScript RT Master Mix (TaKaRa). The part of the coding sequence containing the mutation site was amplified by PCR using cDNA as a template, GoTaq DNA polymerase (Promega), and the *OsABCC1*_cDNA_F and *OsABCC1*_cDNA_R primers (Table S3). The band corresponding to each splicing variant was extracted using a FastGene Gel/PCR Extraction Kit (Nippon Genetics) and subjected to Sanger sequencing using the *OsABCC1*_cDNA_Splicing_F primer (Table S3).

### 2.8 Quantification of mRNA Accumulation

Roots of two‐week‐old seedlings of the WT and *oshma2‐4* plants were frozen in liquid N_2_ for 1 h. Total RNA was extracted using the NucleoSpin RNA Plant kit (TaKaRa), and first‐strand cDNA was synthesized using PrimeScript RT Master Mix (TaKaRa). Quantitative real‐time PCR (qPCR) was performed using TB Green Premix Ex Taq II (TaKaRa). *OsHMA2* transcript levels were normalized to the *Actin1* gene, and mRNA accumulation was quantified using a standard curve method. The primers used for qPCR are listed in Table S3.

### 2.9 Statistical Analysis

All statistical analyses in this study were performed using R version 4.5.1 and RStudio (R Core Team 2024). Figures were prepared using the “ggplot2” package.

## 3 Results

### 3.1 Thirty‐Eight Mutants Were Isolated Through Screening

**FIGURE 1:**
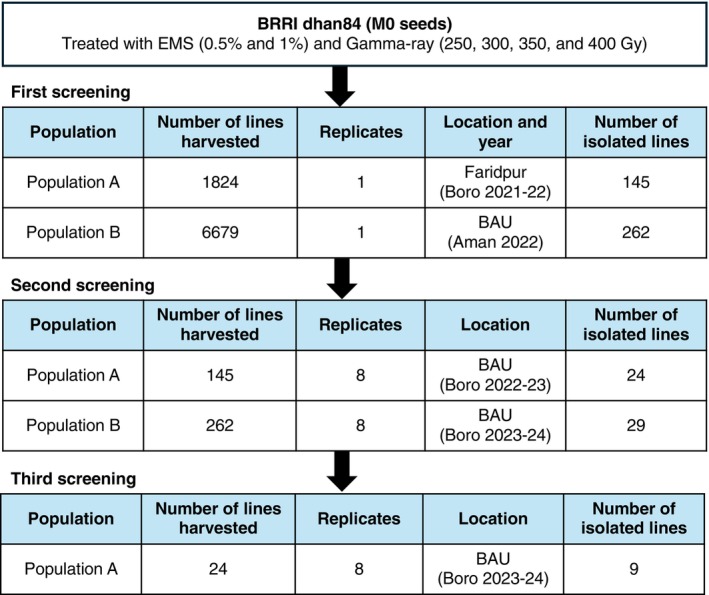
Summary of the mutant screening process. The M_1_ seeds were initially grown in the paddy field of Bangladesh Agricultural University (BAU) during the boro (dry) season (December 2020–May 2021), and three panicles were harvested. From each panicle, one M_2_ seed was germinated and advanced through two separate populations. *Population A:* One M_2_ seed from a single panicle was grown in a farmer's field in Faridpur, an As‐contaminated area, during the boro season (December 2021–May 2022), and five panicles were harvested. *Population B:* One M_2_ seed from a mixture of two panicles was grown in the BAU field during the Aman (monsoon) season (July–November 2022), and five panicles were harvested. Grains from the five panicles of each population were subjected to ionome screening by ICP‐MS to isolate mutants. The selected M_3_ plants from populations A and B were grown in the BAU field during the boro seasons of 2022–2023 and 2023–2024, respectively. The selected M_4_ plants were further grown during the boro season of 2023–2024 at BAU for the third screening.

**FIGURE 2:**
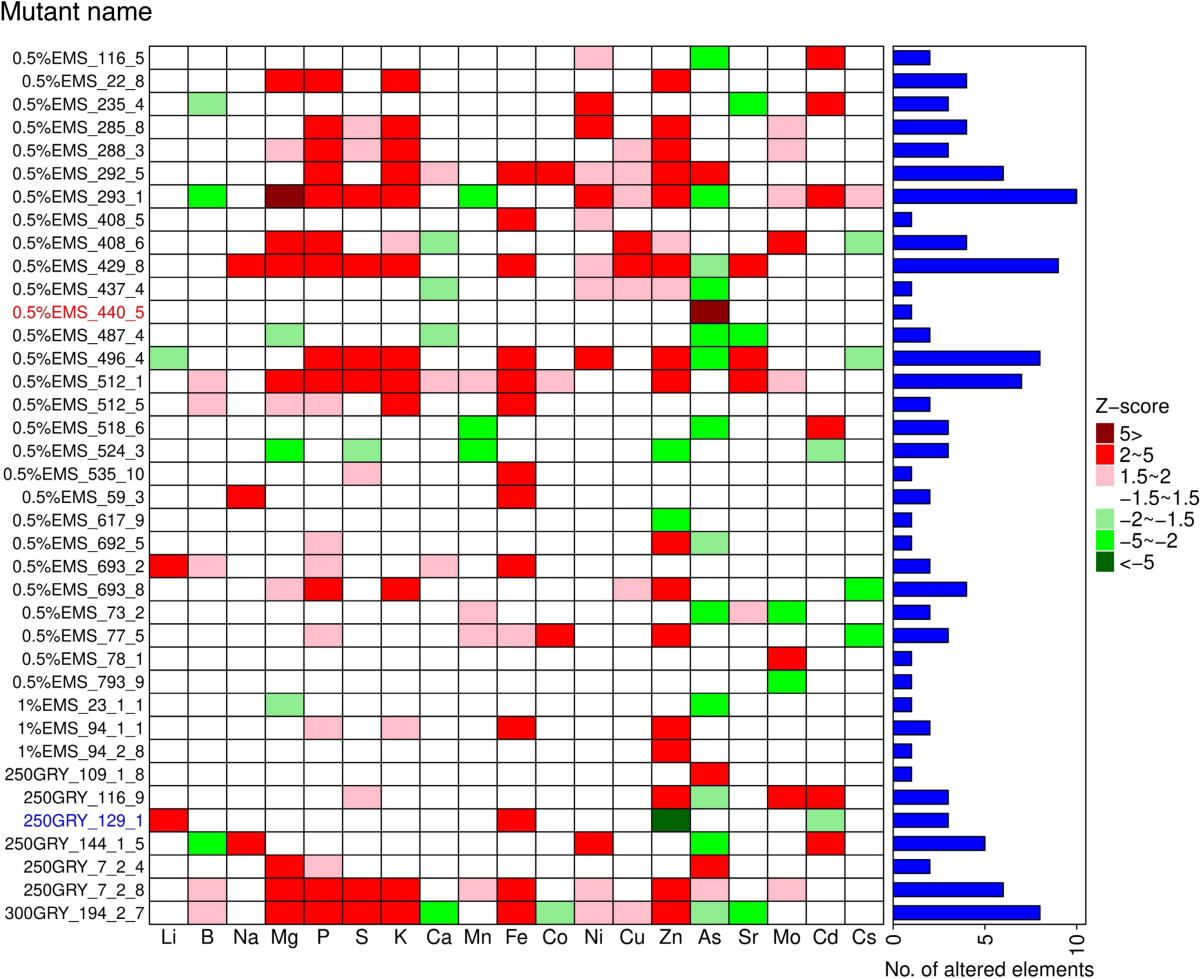
*Z*‐score plot of the isolated 38 mutants. The *Z*‐scores for each element of a mutant were calculated based on the median value of 4–8 plants. The plants were grown at the BAU paddy field in 2024. These 38 mutants include 9 from Population A and 29 from Population B, as shown in Figure 1. The mutants were isolated when the |*Z*‐score| is > 2. The bar plot indicates the number of elements having a |*Z*‐score| > 2. The red text indicates the *osabcc1‐3* mutant, and the blue text indicates the *oshma2‐4* mutant.

To isolate mutants with altered grain concentrations of Zn, Fe, As, and Cd, we grew a total of 8503 M_2_ plants from two populations (A and B) across different fields in Bangladesh and determined the ionome (concentrations of 19 elements) in the brown rice. Based on the robust *Z*‐score (|*Z*‐scores| > 2), 145 lines from population A and 262 lines from population B were isolated in the first screening and subjected to the second screening (Figure 1). After the second screening, 24 lines from population A and 29 lines from population B with reproducible phenotypes were selected for the third screening (Figure 1). Following the third screening of population A, nine reproducible lines were isolated. In total, 38 mutants (nine from the third screening of population A and 29 from the second screening of population B) were obtained from the screening. Of those, 32 mutants exhibited beneficial grain phenotypes (i.e., high Fe, high Zn, low As, and low Cd); three mutants exhibited high As; one mutant exhibited low Zn; and two mutants exhibited other obvious phenotypes, such as high Mo (0.5%EMS_78_1) or low Mo (0.5%EMS_793_9) (Figure 2). To validate our screening process, we selected two mutants for further analysis: 0.5%EMS_440_5 for high As and 250GRY_129_1 for low Zn and Cd (Figure 2, Figure S2).

### 3.2 Element Concentrations in Grains of Beneficial Mutants

**FIGURE 3:**
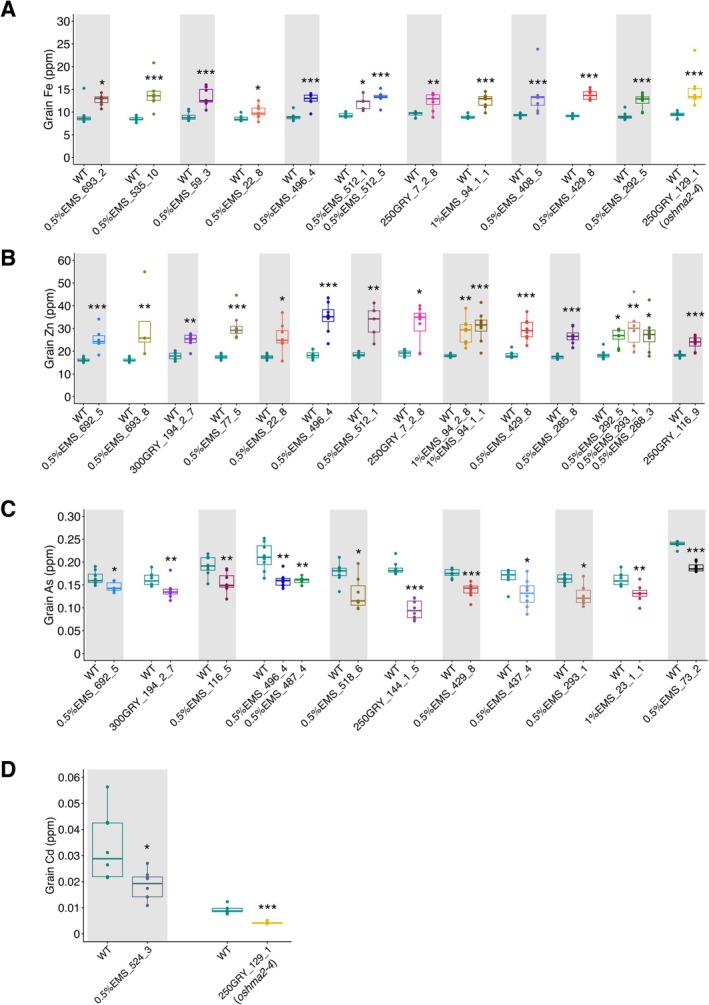
Element concentrations in grains of beneficial mutants. (A) Grain Fe concentration in high‐Fe mutants. (B) Grain Zn concentration in high‐Zn mutants. (C) Grain As concentration in low‐As mutants. (D) Grain Cd concentration in low‐Cd mutants. The WT plants used for comparison were the nearest WT individuals planted beside each mutant. Significant differences were calculated using the Wilcoxon test when comparing a single mutant with WT and the Kruskal‐Wallis test followed by Dunnett's test when comparing multiple mutants with WT. *n* = 4–8; ***p < 0.001, **p < 0.01, *p < 0.05.

To evaluate the change in element concentrations, the element concentrations of mutants were plotted with the WT, BRRI dhan84 (Figure 3). During screening, WT plants were planted at the borders and repeated at regular intervals among the mutant lines (one WT followed by 10 mutant plants). This approach was used to minimize the environmental effects during the comparison of element concentrations. In Figure 3, each mutant was compared to the nearest spatially adjacent WT. Thirteen mutants exhibited significantly higher grain Fe concentrations, with increases of 15.7%–80.6% compared to the WT (Figure 3A). Sixteen mutants had significantly high grain Zn concentrations, with increases of 32.5%–94.0% compared to the WT (Figure 3B). Twelve mutants contained significantly lower grain As, with reductions of 10.5%–48.2% compared to the WT (Figure 3C). Two mutants, 0.5%EMS_524_3 and 250GRY_129_1 (*oshma2‐*4), exhibited significantly lower Cd concentrations in grains, with reductions of 33.0% and 53.1%, respectively, compared to the WT (Figure 3D).

### 3.3 *OsABCC1* Is the Candidate Gene for High As Mutant

**FIGURE 4:**
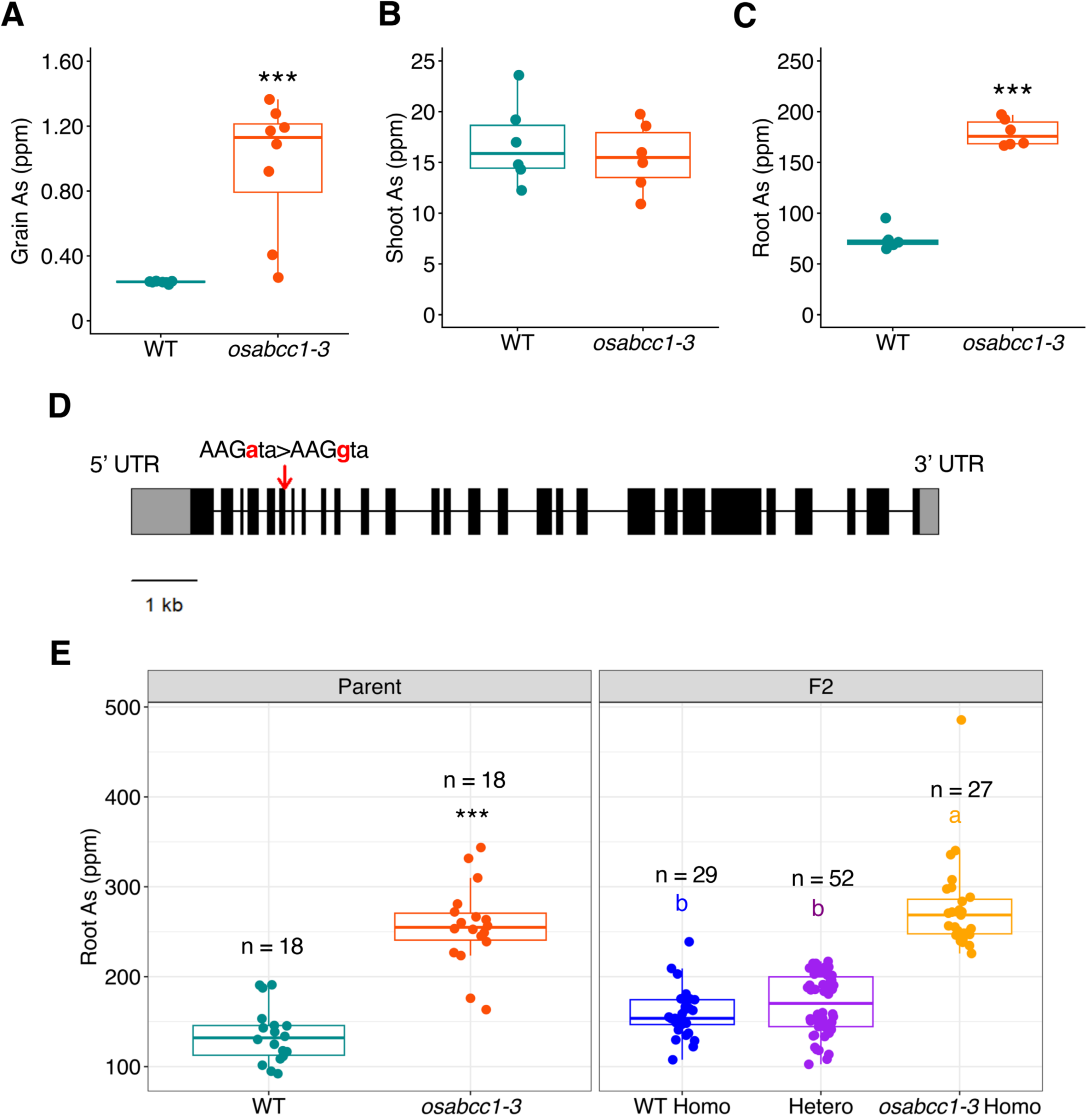
*OsABCC1* is the candidate gene for the high As phenotype in the *osabcc1‐3* mutant. (A) Grain As concentration in WT and *osabcc1‐3* mutant grown at the BAU paddy field in 2024. Data are the same as in Figure 2. *n* = 8, Student's *t*‐test, ***p < 0.001. (B) Shoot As concentration. (C) Root As concentration. Shoot and root As were determined in two‐week‐old plants grown in Kimura B solution with 100 ppb As(III) in the greenhouse (20°C–30°C, sunlight with open air). *n* = 6, Student's *t*‐test, ***p < 0.001. (D) Gene structure of the *OsABCC1* gene and the mutation site in the os*abcc1‐3* mutant. The box and line indicate the exon and intron, respectively. The red arrow indicates the site of mutation in the *osabcc1‐3* mutant. (E) Root As concentration of parents and F_2_ plants. Root As was determined in 2‐week‐old plants grown in Kimura B solution with 100 ppb As(III) in the greenhouse (20°C–30°C, sunlight with open air). The significant differences were calculated using Student's *t*‐test for parents, ***p < 0.001, and Tukey's HSD (*p* < 0.05) for F_2_ genotypes. Letters indicate significant differences. The number of replicates is shown in the figure.

To validate our screening process, we focused on the mutant 0.5%EMS_440_5 (hereafter *osabcc1‐3*), which exhibited four times higher grain As concentrations compared to the WT (Figure 4A). The mutant also showed a similar As concentration in hydroponically grown shoots (Figure 4B), while root As was 2.3 times higher in the mutant compared to the WT (Figure 4C). We further tested As sensitivity under different As (III) concentrations, as it is the major form of As in paddy fields (anaerobic conditions) (Das and Biswas 2022). No significant differences were observed in shoot and root length under lower As levels (10–100 ppb); however, both shoot and root growth were markedly reduced in the mutant at As concentrations ranging from 500 to 2000 ppb As (Figure S3).

Identification of the causal gene responsible for a mutant phenotype using a genome‐wide approach requires several years. To accelerate the identification of the gene responsible for high As in the *osabcc1‐3* mutant, we determined the whole genome sequence of the mutant by next‐generation sequencing (NGS) and specifically examined the DNA sequences of *OsABCC1* or *OsPCS1*, as disruptions of these genes have been shown to result in high As accumulation in grains and increased As sensitivity (Song et al. 2014; Hayashi et al. 2017). We found that the *osabcc1‐3* mutant carries a point mutation at the exon‐intron junction of the *OsABCC1* gene (Figure 4D). To confirm whether this mutation is responsible for the phenotype of the *osabcc1‐3* mutant, we examined the correlation between genotype (mutation in *osabcc1‐3*) and phenotype (root As) in F_2_ crosses between WT and the *osabcc1‐3* mutant. The homozygous *osabcc1‐3* lines showed higher root As concentrations than the homozygous WT, which is similar to the heterozygous lines (Figure 4E). These results suggest that *OsABCC1* is the candidate gene responsible for high As in the *osabcc1‐3* mutant.

To further investigate whether this mutation caused a splicing error in the mutant, we performed PCR with primers flanking the mutation site using cDNA as a template. A single 201‐bp band was observed in the WT sample, while four bands, each larger than 201 bp, were observed in the *osabcc1‐3* mutant, indicating the production of four splicing variants (Figure S4A). To determine the sequence of the splicing variants, we extracted the bands separately and applied for Sanger sequencing. The sequencing results revealed that variant 2, the major band in the mutant, retained the full‐length 106 bp of the 6th intron, while variant 3 and variant 4 retained 32 and 8 bp from the 6th intron, respectively (Figure S4B). All variants produced a premature stop codon (Figure S4C), which could disrupt gene function. Taken together with the mutant phenotype, these results suggest that *OsABCC1* is the candidate gene. Furthermore, the identification of the mutant with the reported gene validates our screening process.

### 3.4 *OsHMA2* Is the Candidate Gene for Low Zn and Cd in oshma2‐4 Mutant

**FIGURE 5:**
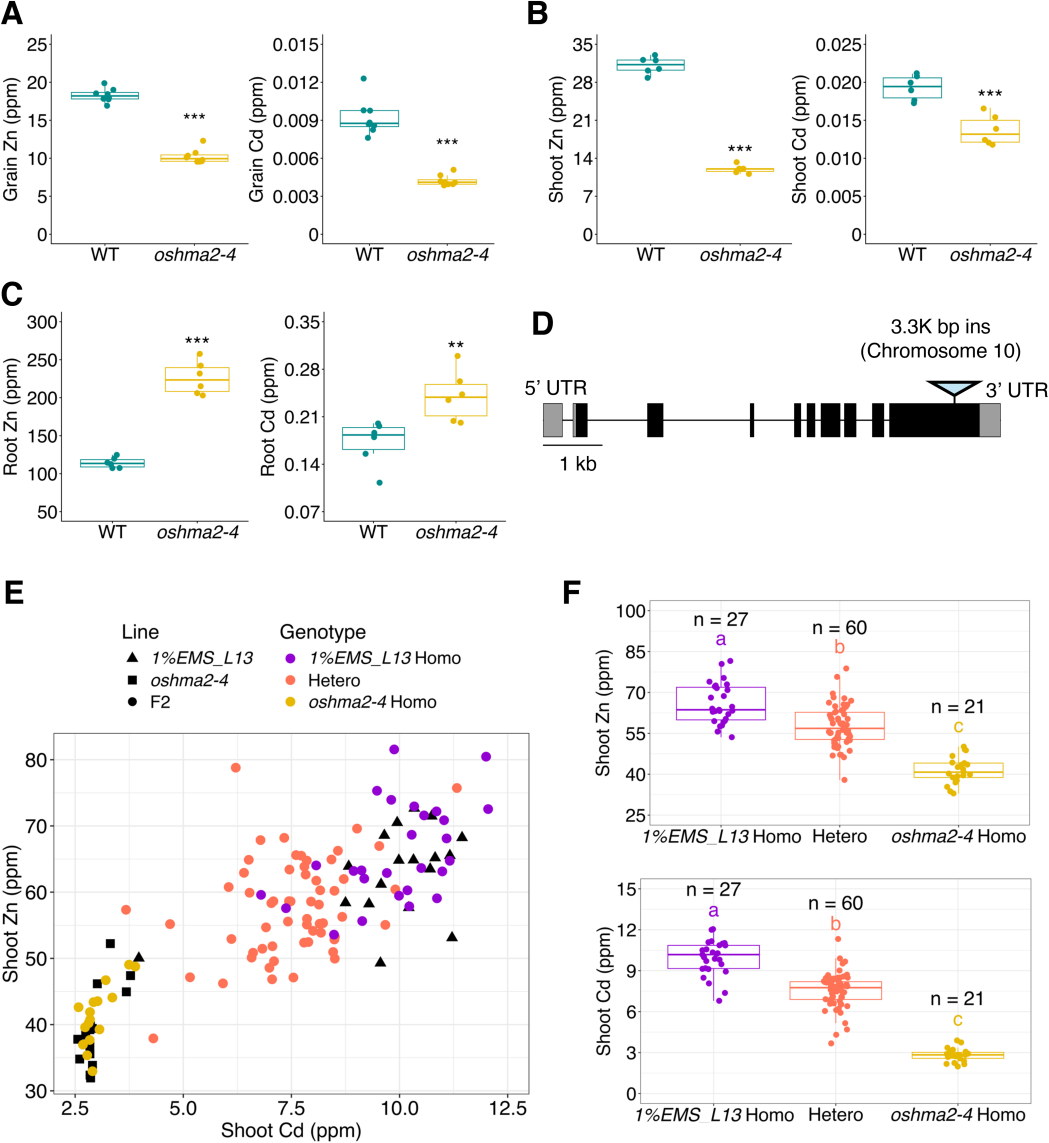
*OsHMA2* is the candidate gene for the phenotypes in the *oshma2‐4* mutant. (A) Zn and Cd concentrations in grain. The plants were grown at the BAU paddy field in 2024. Data are the same as in Figure 2 and Cd concentration in Figure 3. *n* = 8, Student's *t*‐test, ***p < 0.001. (B) Zn and Cd concentrations in shoot. (C) Zn and Cd concentrations in root. Shoot and root concentrations were determined in 2‐week‐old plants grown in Kimura B solution in the greenhouse (20°C–30°C, sunlight with open air). *n* = 6, Student's *t*‐test, ***p < 0.001, **p < 0.01. (D) Gene structure of *OsHMA2* and the mutation site in the *oshma2‐4* mutant. The box and line indicate the exon and intron, respectively. The triangle indicates the site of mutation in the gene. (E) A correlation plot between Zn and Cd concentrations in shoots for each genotype in the F_2_ population. F_2_ plants were grown together with the parental line in Kimura B solution with 10 ppb Cd. (F) Boxplot of different F_2_ genotypes in (E). Significant differences were calculated using Tukey's HSD (*p* < 0.05). Letters indicate significant differences. The number of replicates is shown in the figure.

To identify the mutation responsible for the beneficial phenotype, low Cd in grain, we studied the *oshma2‐4* mutant. The *oshma2‐4* mutant exhibited lower concentrations of both Cd and Zn in grain compared to the WT (Figure 5A). Similarly, Cd and Zn concentrations in the hydroponically grown shoot were lower in the *oshma2‐4* mutant (Figure 5B), while concentrations were higher in the roots of the *oshma2‐4* mutant compared to the WT (Figure 5C).

Following a similar strategy to that used for the *osabcc1‐3* mutant, we determined the whole‐genome sequence of the *oshma2‐4* mutant by NGS to identify the mutation responsible for the low Cd and Zn accumulation. We focused on the sequences of *OsZIP7* and *OsHMA2*, as disruptions of these genes lead to low Cd and Zn in rice grain (Yamaji et al. 2013; Tan et al. 2019). The sequence analysis by NGS revealed that the *oshma2‐4* mutant has a 3.3 kb insertion from chromosome 10 in the *OsHMA2* gene (Figures 5D and S5), a gene responsible for root‐to‐shoot translocation of Cd and Zn in rice (Takahashi et al. 2012). To investigate whether *OsHMA2* is responsible for the phenotype in the *oshma2‐4* mutant, we examined the correlation between genotype (mutation in *oshma2‐4*) and Cd and Zn concentrations in the shoot of F_2_ crosses between the *oshma2‐4* mutant and the *1%EMS_L13* mutant. The plants were hydroponically grown, and the shoots were applied for ICP‐MS analysis and genotyping. The homozygous *oshma2‐4* lines showed lower Cd and Zn concentrations than the WT homozygous and heterozygous lines (Figure 5E,F). The heterozygous lines displayed intermediate concentrations between the WT and mutant type, indicating that the low Cd and Zn phenotype is semi‐dominant. These results suggest that *OsHMA2* is the candidate gene responsible for low Cd and Zn in the *oshma2‐4* mutant.

To assess the effect of the mutation, we determined the *OsHMA2* cDNA sequence of *oshma2‐4*. We designed several primers, including one located after the putative stop codon (Figure S6A), and performed PCR followed by Sanger sequencing. When primers were designed in exon 2 and exon 9 (A and B in Figure S6A, B), a similar PCR product was obtained for both WT and *oshma2‐*4, indicating that the mutation did not affect transcript length or splicing in this region. In contrast, when primers were designed before the insertion site and after the putative stop codon (C and D in Figure S6A, B), a PCR fragment was observed only in *oshma2‐4*, indicating that this stop codon is present in the mutant. This result indicates that the mutated‐type *OsHMA2* protein has a premature stop codon (Figure S6C, D) near the C terminus, where no domain and transmembrane are present (Figure S6E). We also quantified *OsHMA2* transcript levels in *oshma2‐4* using qPCR primers located in exon 2, distant from the mutation site. Transcript quantification showed similar levels of *OsHMA2* mRNA between WT and *oshma2‐4* (Figure S6F), suggesting that the truncated protein is expressed in the mutant.

### 3.5 *o* *s* *h* *m* *a* *2‐4* Mutant Has Potential for Breeding Low‐Cd Rice

**FIGURE 6:**
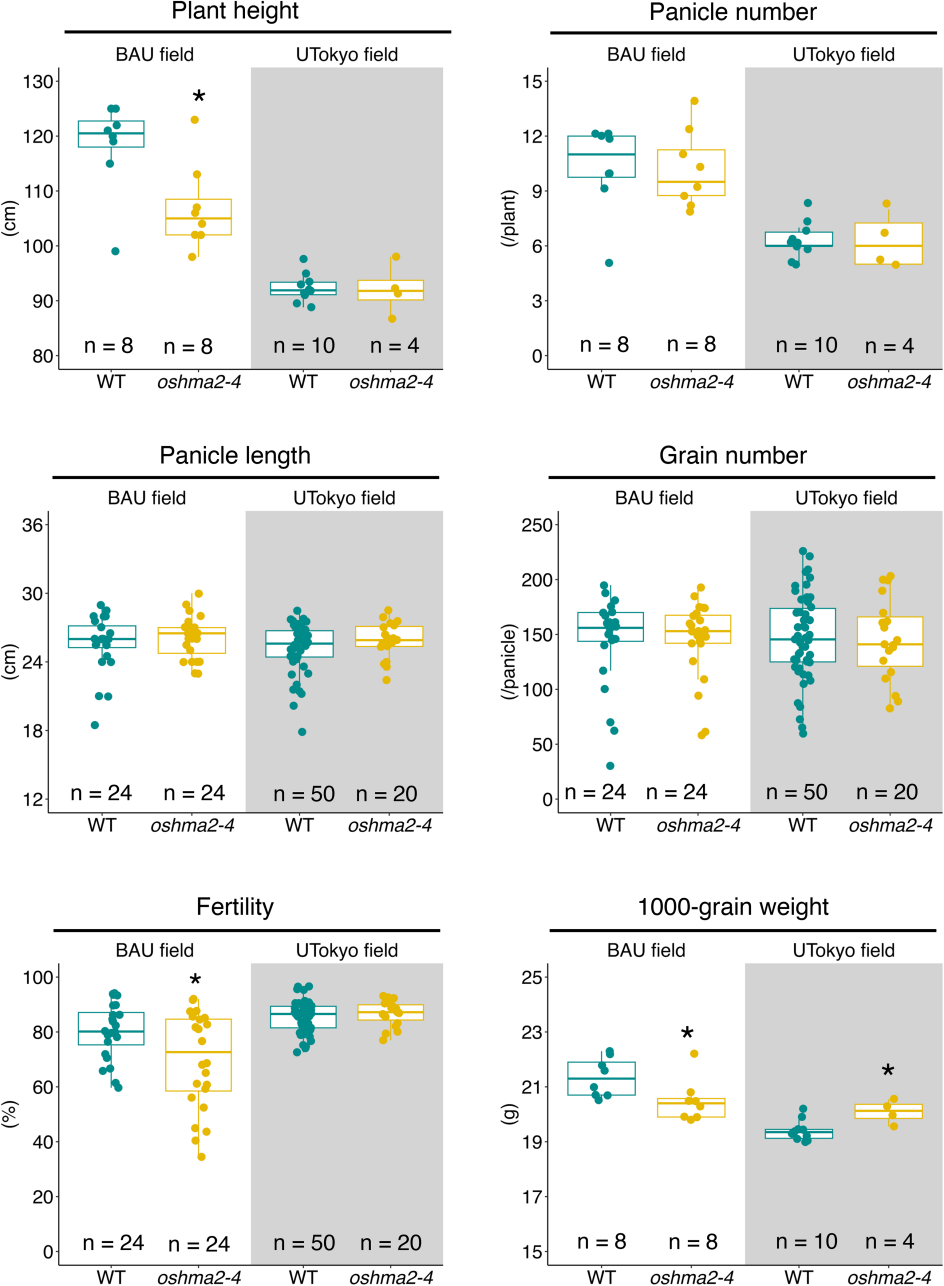
Growth and yield performance of the *oshma2‐4* mutant in the field. Plants were grown at the Bangladesh Agricultural University (BAU) in 2024 and UTokyo paddy fields in 2024. Data were recorded from 8 plants for both WT and *oshma2‐4* at the BAU field and 10 plants for WT and 4 plants for *shma2‐4* at the UTokyo field. The data of panicle length, grain number, and fertility were recorded from 3 panicles and 5 panicles of each plant at the BAU field and the UTokyo field, respectively. The significant differences were calculated using Student's *t*‐test. *p < 0.05. The number of replicates is shown in the figure.

To evaluate whether the *oshma2‐4* mutant can be used for breeding low‐Cd rice, we grew the mutant with WT plants in Bangladesh and Japan and measured the growth and yield parameters, such as plant height, panicle number, panicle length, grain number, fertility, and 1000‐grain weight. In the BAU field (Bangladesh), the *oshma2‐4* mutant exhibited significantly reduced plant height, fertility, and 1000‐grain weight compared to the WT (Figure 6). In contrast, no significant decrease was observed between WT and *oshma2‐4* mutant plants in the UTokyo field (Japan) (Figure 6), suggesting that the *oshma2‐4* mutant can be used for breeding low‐Cd rice.

### 3.6 oshma2‐4 Mutant Is Sensitive to High Cd

Because the *oshma2‐4* mutant accumulates low levels of Cd and Zn, we evaluated its growth response under low Zn and high Cd conditions to determine whether it can grow under these stressed conditions. For the low Zn experiment, we grew both WT and *oshma2‐4* plants using Kimura B solution with or without Zn for 3 weeks. Growth was similar between the WT and the mutant, except for the shoot length under normal Kimura B (0.15 μM) (Figure S7).

**FIGURE 7:**
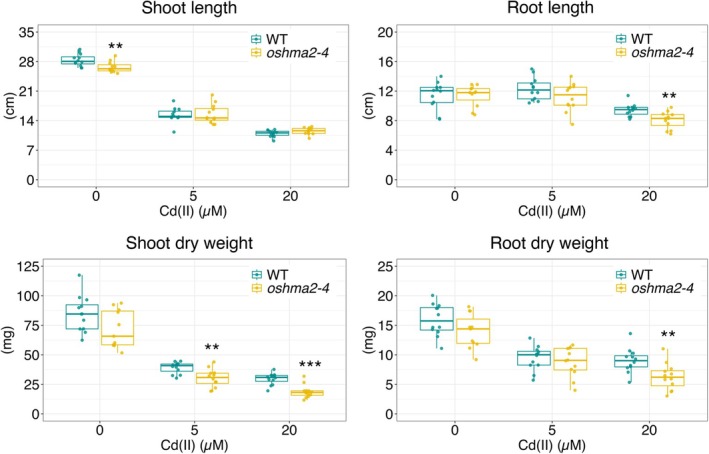
Cd sensitivity of the *oshma2‐4* mutant. Plants were grown in Kimura B solution with different Cd doses for 3 weeks in the greenhouse (20°C–30°C, sunlight with open air). *n* = 10–12, Student's *t*‐test, ***p < 0.001, **p < 0.01, *p < 0.05.

For the high Cd experiment, both WT and *oshma2‐4* plants were grown in Kimura B solution supplemented with different Cd concentrations. Plant growth was similar under 0 and 5 μM Cd, but at 20 μM Cd, the *oshma2‐4* mutant exhibited significantly lower shoot and root biomass and shorter root length compared to the WT (Figure 7). These results suggest that *oshma2‐4* is sensitive to high Cd concentrations and that *OsHMA2* contributes to high‐Cd tolerance.

## 4 Discussion

This study presents the first large‐scale ionome profiling of brown rice from a mutagenized population of the *indica* rice, providing valuable genetic resources for improving grain elemental composition. In a similar study, Tanaka et al. (2016) conducted a large‐scale ionome screening in an EMS‐mutagenized population of the Japanese rice cultivar “Hitomebore” and isolated a beneficial low‐Cd line. Mutagenesis provides the advantage of generating single‐locus variations with phenotypic effects, which facilitates the identification of causal genes and the development of marker‐assisted breeding materials. The 32 beneficial mutants isolated in this study, including those with high Zn and Fe and low As and Cd concentrations in grain (Figure 3), represent valuable genetic resources for improving the nutritional quality and safety of rice through marker‐assisted breeding.

The *OsABCC1* gene encodes a tonoplast‐localized ABC transporter responsible for sequestering As into vacuoles, thereby limiting its accumulation in grains (Song et al. 2014). Knockout or EMS mutants of *OsABCC1* have been shown to exhibit elevated grain As levels and increased As sensitivity (Song et al. 2014; Hayashi et al. 2017; Yang et al. 2025). Consistent with these reports, the *osabcc1‐3* mutant isolated in this study showed both high grain As accumulation and increased As sensitivity (Figures 4A and S3). Taken together with the effect of the mutation in splicing (Figures 4D and S4) and the correlation between genotype and phenotype (Figure 4E), *OsABCC1* is likely the causal gene. While this allele is not beneficial for breeding of low As, it could be beneficial for phytoremediation of As in soil.

The *OsHMA2* gene encodes a plasma‐membrane P_1B_‐type ATPase involved in xylem loading and root‐to‐shoot translocation of Zn and Cd (Satoh‐Nagasawa et al. 2012). Knockout mutants of *OsHMA2* exhibit reduced Zn and Cd accumulation in shoots and grains but elevated levels in roots (Satoh‐Nagasawa et al. 2012; Takahashi et al. 2012; Yamaji et al. 2013). In line with these findings, the *oshma2‐4* mutant identified in this study exhibited low Zn and Cd concentrations in shoots and grains, with high concentrations in roots (Figure 5). Taken together with the effect of mutation in the transcript (Figure S6F) and the correlation between genotype and phenotype (Figure 5E,F), *OsHMA2* is likely the causal gene.

Under field conditions, *oshma2‐4* did not show growth inhibition in the UTokyo field; however, in the BAU field, *oshma2‐4* showed reduced plant height, fertility, and 1000‐grain weight compared with the WT (Figure 6). This result raises two questions. Firstly, why did the *oshma2‐4* mutant grow normally in UTokyo, while the *Tos17* mutants exhibit impaired growth and fertility under field conditions (Yamaji et al. 2013)?

A primary factor that could explain this discrepancy is that the mutated *OsHMA2* in *oshma2‐4* likely retains partial function. Unlike the *Tos17* mutants, which exhibit no detectable transcripts (Yamaji et al. 2013), *oshma2‐4* maintained transcript levels similar to those of the WT. Because the insertion is located in the C‐terminus, *oshma2‐4* is predicted to produce a truncated OsHMA2 protein (925 amino acids) that retains the HMA domain and all transmembrane regions (Figure S6E,F). This hypothesis is supported by previous research. Satoh‐Nagasawa et al. (2012) demonstrated that truncated *Tos17* mutants of *OsHMA2*, specifically *oshma2‐1* (lacking the last four transmembrane domains, consisting of 316 amino acids) and *oshma2‐2* (lacking the C‐terminus, consisting of 740 amino acids), remained functional in yeast. Notably, the *oshma2‐2* mutant plants grew similarly to the WT. Furthermore, the authors showed that while the C‐terminus of *OsHMA2* is crucial for Cd translocation in rice, proteins lacking this region can still translocate Zn to some extent. Taken together, the truncated *OsHMA2* (925 amino acids) in *oshma2‐4* could be expressed and partially functional as a Zn transporter, allowing the plants to avoid severe growth defects under certain field conditions. Another possibility is the difference in genetic background. The *Tos17 OsHMA2* mutant is in a *japonica* (Nipponbare) background (Yamaji et al. 2013). In several GWAS analyses, loci responsible for Zn (grain and shoot) have been identified (Norton et al. 2014; Cu et al. 2021; Liu et al. 2021). The combination of these loci and the *OsHMA2* mutation may lead to the difference in growth.

The second question is why the growth inhibition of *oshma2‐4* was observed only in the BAU field but not in the UTokyo field or in our hydroponic experiments using Kimura B solution. This discrepancy may be attributed to the low Zn availability in the BAU soil combined with the effects of long‐term cultivation. The BAU soil is classified as low‐Zn soil based on its available Zn concentration, and plants were cultivated with the recommended fertilizer dose of 1.5 kg Zn ha^−1^ (Ahmmed et al. 2018). However, in another study, Afrin et al. (2022) showed that rice growth and yield significantly increased with 10 kg Zn ha^−1^ compared to no Zn and 5 kg Zn ha^−1^ in a different plot of the BAU field, which is also classified as low‐Zn soil. These findings suggest that BAU soil is Zn‐deficient, and 1.5 kg Zn ha^−1^ was not enough for normal growth of the *oshma2‐4* mutant.

Furthermore, the duration of cultivation likely played a critical role. While no phenotypic differences were observed during the short‐term (three‐week) hydroponic screening in Kimura B solution, the cumulative impact of Zn deficiency over the entire growth stage in the BAU field manifested as reduced plant height, fertility, and 1000‐grain weight. These results suggest that the partial function of *OsHMA2* in *oshma2‐4* may be sufficient for early development or under Zn‐sufficient conditions but is inadequate to support full reproductive development in Zn‐deficient field environments.

The *oshma2‐4* mutant showed sensitivity to high Cd levels under hydroponic conditions (Figure 7). The available Cd in soil solution is generally low, ranging from less than 0.1 to 5 ppb in uncontaminated soils to 160 ppb in paddy soils near Cd pollution sites, and is influenced by several factors such as pH, redox potential, organic matter, and competing ions (Akahane et al. 2013; Smolders and Mertens 2013). In hydroponic culture, the *oshma2‐4* mutant showed similar growth to the WT under 5 μM Cd (equivalent to 562 ppb), whereas a significant growth reduction was observed under 20 μM Cd (equivalent to 2.25 ppm) (Figure 7). These results suggest that this allele can tolerate Cd concentrations several orders of magnitude higher than those typically found in contaminated soils and can be used for cultivation in Cd‐contaminated soils. Thus, *oshma2‐4* represents a new functional allele that can serve as a low‐Cd donor for breeding programs, even in Cd‐contaminated soils.

Collectively, the *oshma2‐4* mutant would be suitable low‐Cd breeding material for normal paddy fields in Bangladesh and for low Zn‐deficient soils provided with appropriate Zn fertilization. Given its low Zn content, Zn deficiency could be compensated by introducing high‐Zn alleles through crossing or applying agronomic Zn biofortification strategies such as foliar Zn spray. Future studies should aim to genetically decouple Zn and Cd transport to achieve both safety and nutritional enhancement.

## 5 Conclusion

In conclusion, large‐scale ionomic screening of a mutagenized BRRI dhan84 population isolated 38 mutants with distinct grain phenotypes, including two mutants with known functional disruptions in *OsABCC1* and *OsHMA2*. The identification of these new alleles not only validates the ionomic screening process but also demonstrates the potential of identifying causal mutations (candidate genes) by whole‐genome sequencing and segregation analysis. The *oshma2‐4* mutant represents a promising low‐Cd resource for breeding. Future research integrating transcriptomic, physiological, and field evaluations of the isolated mutants will facilitate the development of rice cultivars that are both nutritionally enriched and environmentally safe for human consumption.

## Author Contributions

Toru Fujiwara and Takehiro Kamiya contributed to the study conception; Shihab Uddin, Md. Rafiqul Islam, Mirza Mofazzal Islam, and Md. Abdul Kader prepared the mutant populations; Shihab Uddin and Takehiro Kamiya contributed to ICP‐MS analysis and mutant selection; Takehiro Kamiya contributed to the NGS analysis and selection of candidate genes; Shihab Uddin performed hydroponic and field experiments; Shihab Uddin drafted the manuscript under the supervision of Takehiro Kamiya; Md. Rafiqul Islam, Toru Fujiwara, and Takehiro Kamiya edited the manuscript. All authors read and approved the final manuscript.

## Funding

This research was supported by the Japan Science and Technology Agency (JST)/Japan International Cooperation Agency (JICA) under the Science and Technology Research Partnership for Sustainable Development (SATREPS) project, grant number JPMJSA2107.

## Conflicts of Interest

The authors declare no conflicts of interest.

## Supporting information


**Figure S1:** Zinc and cadmium concentrations of the WT and 1%EMS_L13 mutant used for crossing. (A) Grain Zn and Cd concentrations of WT and 1%EMS_L13 plants grown in pots in the greenhouse. 10 ppb Cd was added every 3 weeks during the whole growth period. (B) Shoot Zn and Cd concentrations of the 2‐week‐old plants of WT and 1%EMS_L13 grown in commercial full‐nutrient soil (Honens soil, HONEN AGRI) in the greenhouse (20°C–30°C, sunlight with open air). The number of replicates is shown in the figure. The *p*‐values in the figure were calculated using Student's *t*‐test.
**Figure S2:** Z‐score plot of the osabcc1‐3 and oshma2‐4 mutants in the second screening. (A) Z‐score plot of osabcc1‐3 mutant. (B) Z‐score plot of the oshma2‐4 mutant. Red boxes in the figure indicate the plants selected for planting in 2024 at the Bangladesh Agricultural University (BAU) field.
**Figure S3:** Arsenic sensitivity of osabcc1‐3 mutant. (A) Shoot length of 2‐week‐old plants. (B) Root length of 2‐week‐old plants. WT and osabcc1‐3 mutant were grown in Kimura B solution with different As concentrations in the greenhouse (20°C–30°C, sunlight with open air). *n* = 6, Tukey's HSD (*p* < 0.05). Letters indicate significant differences.
**Figure S4:** Splicing variants in osabcc1‐3 mutant. (A) Gel image showing splicing variants in the osabcc1‐3 mutant using a cDNA template. (B) Sequence of the PCR product in WT and osabcc1‐3 mutant by Sanger sequencing. Red text indicates the mutation point in the osabcc1‐3 mutant. The gray shade indicates the intron sequence in WT. The underlined sequence indicates a stop codon. Sequencing of Variant 1 failed. (C) Amino acid sequence of (B). * indicates the stop codon. The number after each variant's amino acid sequence indicates the predicted total size (in amino acids) of the protein produced by each variant.
**Figure S5:** Schematic diagram of the insertion site in the OsHMA2 gene in the oshma2‐4 mutant. (A) Connection points of chromosomes 6 and 10 and their positions in the genome. (B) Sequence of the junction 1. (C) Sequence of the junction 2.
**Figure S6:** Effect of insertion in OsHMA2 transcript. (A) Schematic representation of the positions of primers used for PCR. The arrow indicates the position of each primer. (B) Gel image of PCR products obtained using the primers in (A) with a cDNA as the template. (C) cDNA sequence including junction 1 in WT and oshma2‐4. Light blue and light peach shading indicate sequences derived from chromosomes 6 and 10, respectively. (D) Predicted amino acid sequence from the sequence shown in (C). * indicates the stop codon. (E) Schematic representation of OsHMA2 protein in WT and oshma2‐4 mutant based on the UniProt database (https://www.uniprot.org/uniprotkb/A3BF39/entry#sequences). The purple box indicates the HMA domain, the gray boxes indicate transmembrane regions, and the red triangle marks the insertion site in oshma2‐4. The light peach line indicates the inserted amino acids. (F) Relative OsHMA2 mRNA accumulation. The mRNA accumulation of OsHMA2 was determined in the roots of 2‐week‐old plants grown in a growth chamber (30°C, 76% relative humidity, 16/8 h light/dark cycle, and photosynthetic photon flux density of 690 μmol m^−2^ s^−1^). The mRNA accumulation level of OsHMA2 was normalized to the rice Actin1 gene (mean ± SE; *n* = 6). The significant differences were calculated using Student's *t*‐test (*p* < 0.05).
**Figure S7:** Growth performance of the oshma2‐4 mutant under different Zn concentrations in Kimura B for 3 weeks. *n* = 11–12, Student's *t*‐test, ** *p* < 0.01. The data of Zn (0.15 μM) is the same as in Figure 7 (Cd 0 μM). The plants were grown in the greenhouse (20°C–30°C, sunlight with open air).
**Table S1:** Nutrient composition of the Kimura B solution.
**Table S2:** The certified values, measured values, and recovery rates of elements in the reference material (SRM 1573a, Tomato Leaves; NIST).
**Table S3:** Primers used in this study.

## Data Availability

The data that support the findings of this study are available on request from the corresponding author. The data are not publicly available due to privacy or ethical restrictions.
