# Supplementary material for: Ionomic Screening of BRRI dhan84 Mutagenized Population Identifies Candidate Genes Underlying High Arsenic and Low Zinc/Cadmium Accumulation
Source: Physiol Plant. 2026 Jun 25;178(4):e70980. doi: 10.1111/ppl.70980 (PMC13296837; doi:10.1111/ppl.70980)
Supplement: Supplementary file 1 — Figure S1: Zinc and cadmium concentrations of the WT and 1%EMS_L13 mutant used for crossing. (A) Grain Zn and Cd concentrations of WT and 1%EMS_L13 plants grown in pots in the greenhouse. 10 ppb Cd was added every 3 weeks during the whole growth period. (B) Shoot Zn and Cd concentrations of the 2‐week‐old plants of WT and 1%EMS_L13 grown in commercial full‐nutrient soil (Honens soil, HONEN AGRI) in the greenhouse (20°C–30°C, sunlight with open air). The number of replicates is shown in the figure. The p‐values in the figure were calculated using Student's t‐test. Figure S2: Z‐score plot of the osabcc1‐3 and oshma2‐4 mutants in the second screening. (A) Z‐score plot of osabcc1‐3 mutant. (B) Z‐score plot of the oshma2‐4 mutant. Red boxes in the figure indicate the plants selected for planting in 2024 at the Bangladesh Agricultural University (BAU) field. Figure S3: Arsenic sensitivity of osabcc1‐3 mutant. (A) Shoot length of 2‐week‐old plants. (B) Root length of 2‐week‐old plants. WT and osabcc1‐3 mutant were grown in Kimura B solution with different As concentrations in the greenhouse (20°C–30°C, sunlight with open air). n = 6, Tukey's HSD (p < 0.05). Letters indicate significant differences. Figure S4: Splicing variants in osabcc1‐3 mutant. (A) Gel image showing splicing variants in the osabcc1‐3 mutant using a cDNA template. (B) Sequence of the PCR product in WT and osabcc1‐3 mutant by Sanger sequencing. Red text indicates the mutation point in the osabcc1‐3 mutant. The gray shade indicates the intron sequence in WT. The underlined sequence indicates a stop codon. Sequencing of Variant 1 failed. (C) Amino acid sequence of (B). * indicates the stop codon. The number after each variant's amino acid sequence indicates the predicted total size (in amino acids) of the protein produced by each variant. Figure S5: Schematic diagram of the insertion site in the OsHMA2 gene in the oshma2‐4 mutant. (A) Connection points of chromosomes 6 and 10 and their positions in the geno [file PPL-178-e70980-s001.pdf]

Supplementary materials

Ionomic screening of BRRI dhan84 mutagenized population identifies candidate genes underlying high arsenic and low zinc/cadmium accumulation

Shihab Uddin[1,2], Md. Rafiqul Islam[2], Mirza Mofazzal Islam[3], Md. Abdul Kader[4], Toru Fujiwara[1], and Takehiro Kamiya[1,*]

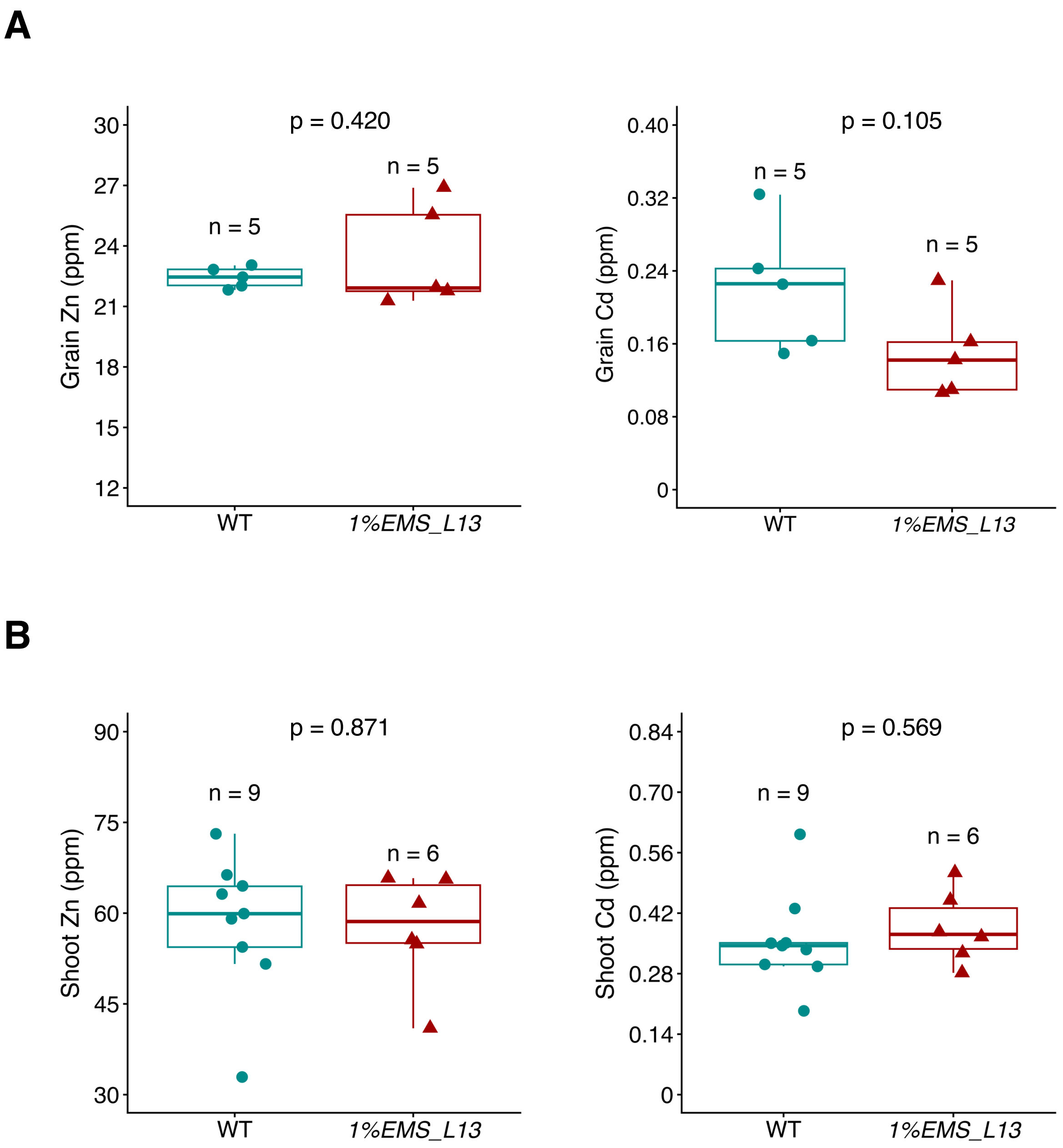

Figure S1 Zinc and cadmium concentrations of the WT and *1%EMS_L13* mutant used for crossing. (A) Grain Zn and Cd concentrations of WT and *1%EMS_L13* plants grown in pots in the greenhouse. 10 ppb Cd was added every three weeks during the whole growth period. (B) Shoot Zn and Cd concentrations of the two-week-old plants of WT and *1%EMS_L13* grown in commercial full-nutrient soil (Honens soil, HONEN AGRI) in the greenhouse (20–30° C, sunlight with open air). The number of replicates is shown in the figure. The p-values in the figure were calculated using Student's *t*-test.

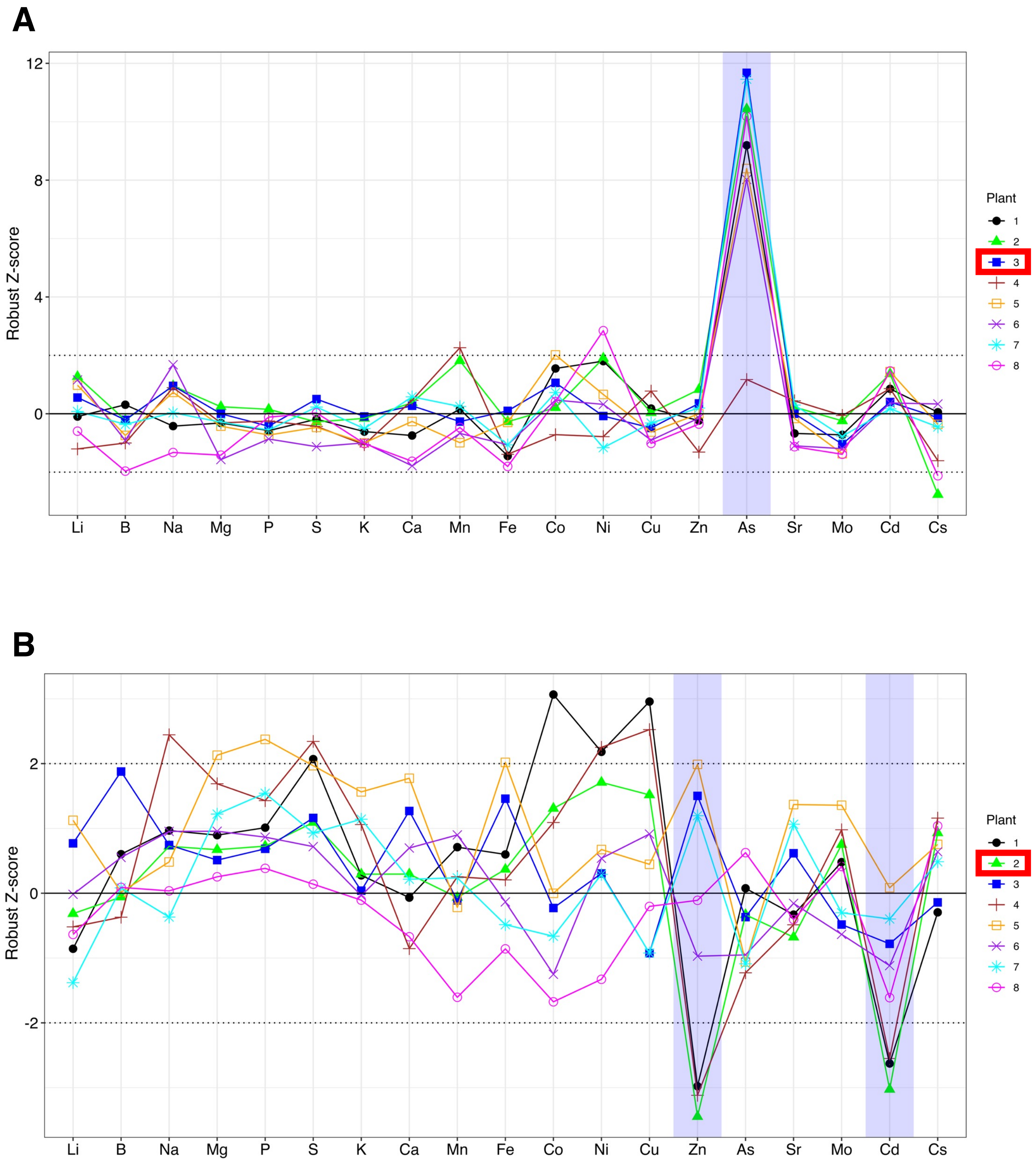

Figure S2 Z-score plot of the *osabcc1-3* and *oshma2-4* mutants in the second screening. (A) Z-score plot of *osabcc1-3* mutant. (B) Z-score plot of *oshma2-4* mutant. Red boxes in the figure indicate the plants selected for planting in 2024 at the Bangladesh Agricultural University (BAU) field.

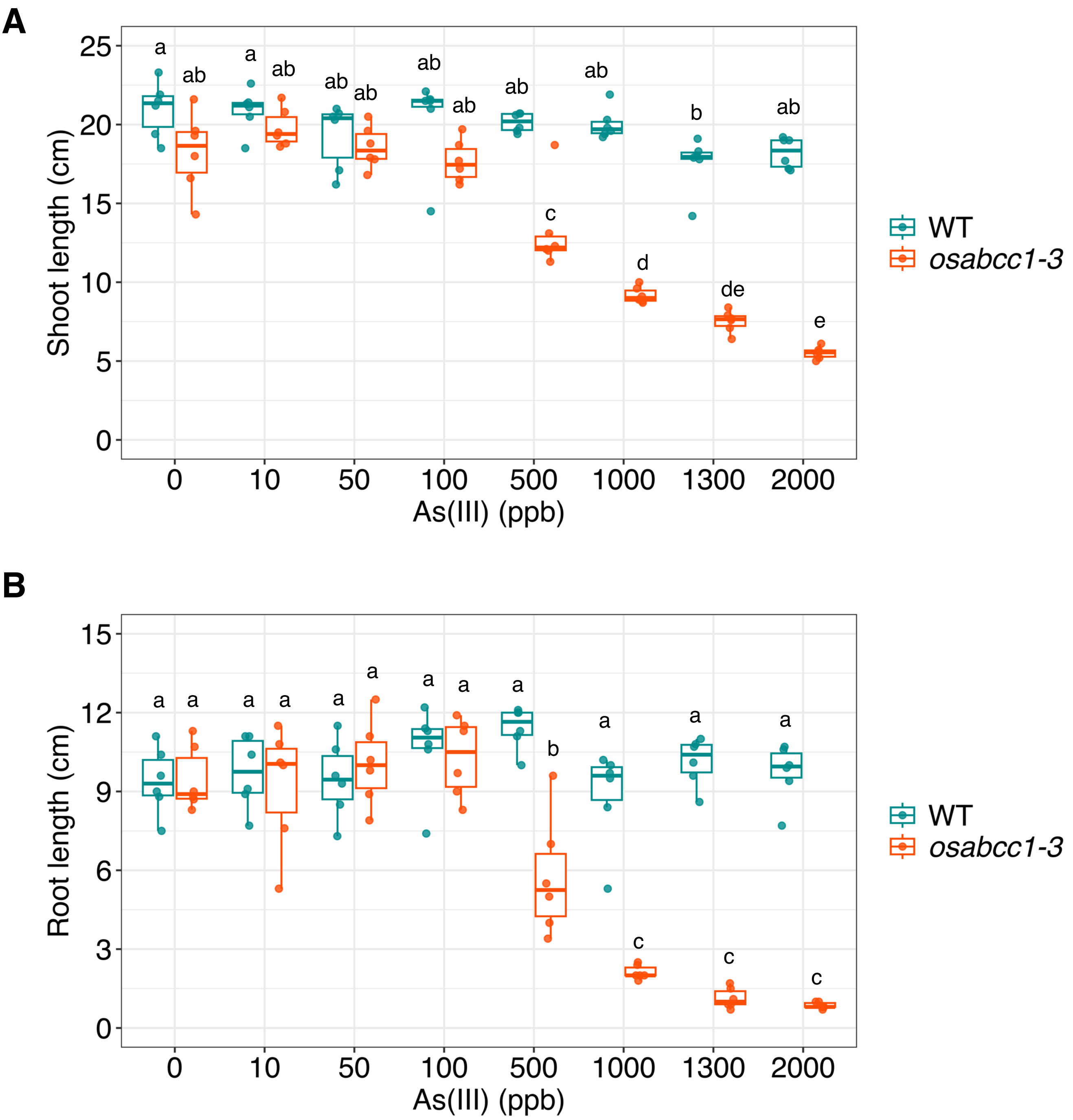

Figure S3 Arsenic sensitivity of *osabcc1-3* mutant. (A) Shoot length of two-week-old plants. (B) Root length of two-week-old plants. WT and *osabcc1-3* mutant were grown in Kimura B solution with different As concentrations in the greenhouse (20–30°C, sunlight with open air). n = 6, Tukey's HSD ($p < 0.05$). Letters indicate significant differences.

A

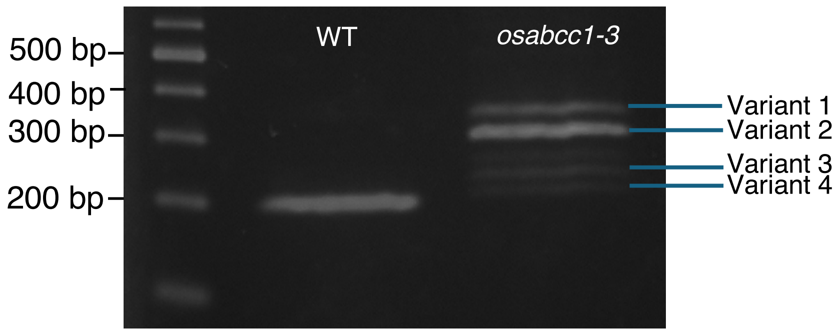

B

```
           861                                            908
       WT: ACCAAAGCCTTGGCTGTTACGGGCTCTGCATAGCAGCCTTGGTGGAAG-----------------------------
Variant 2: ACCAAAGCCTTGGCTGTTACGGGCTCTGCATAGCAGCCTTGGTGGAAGATATATTTGTACAGATGATAGCTATATTT
Variant 3: ACCAAAGCCTTGGCTGTTACGGGCTCTGCATAGCAGCCTTGGTGGAAGATATATTTGTACAGATGATAGCTATATTT
Variant 4: ACCAAAGCCTTGGCTGTTACGGGCTCTGCATAGCAGCCTTGGTGGAAGATATATTT---------------------

                                                                                909
       WT: ----------------------------------------------------------------------------G
Variant 2: TCCAGTAAACCTTTAGGGTGGTCCCTTCTTGCTACATTGTTTGTGTTCACGTAGTGATTGGTTTCATTCATTTTCAG
Variant 3: TCC-------------------------------------------------------------------------G
Variant 4: ----------------------------------------------------------------------------G

           910                                                                  986
       WT: TTTTGGCTAGGAGGATTTTTTAAGATTGGCAATGATGCTTCTCAGTTTGTTGGCCCACTCATATTGAACCTCTTGTT
Variant 2: TTTTGGCTAGGAGGATTTTTTAAGATTGGCAATGATGCTTCTCAGTTTGTTGGCCCACTCATATTGAACCTCTTGTT
Variant 3: TTTTGGCTAGGAGGATTTTTTAAGATTGGCAATGATGCTTCTCAGTTTGTTGGCCCACTCATATTGAACCTCTTGTT
Variant 4: TTTTGGCTAGGAGGATTTTTTAAGATTGGCAATGATGCTTCTCAGTTTGTTGGCCCACTCATATTGAACCTCTTGTT

           987                                                                1061
       WT: GGAGTCTATGCAAAAAGGTGATCCATCTTGGAGCGGTTACATCTATGCTTTCTCAATCTTCGCTGGAGTGTCACT
Variant 2: GGAGTCTATGCAAAAAGGTGATCCATCTTGGAGCGGTTACATCTATGCTTTCTCAATCTTCGCTGGAGTGTCACT
Variant 3: GGAGTCTATGCAAAAAGGTGATCCATCTTGGAGCGGTTACATCTATGCTTTCTCAATCTTCGCTGGAGTGTCACT
Variant 4: GGAGTCTATGCAAAAAGGTGATCCATCTTGGAGCGGTTACATCTATGCTTTCTCAATCTTCGCTGGAGTGTCACT
```

C

```
WT         Reference   287  PKPWLLRALHSSLGGRFWLGGFFKIGNDASQFV 321
osabcc1-3  Variant 2        PKPWLLRALHSSLGGRYICTDDSYIFQ*       315
           Variant 3        PKPWLLRALHSSLGGRYICTDDSYIFRFG*     317
           Variant 4        PKPWLLRALHSSLGGRYICFG*             309
```

Figure S4 Splicing variants in *osabcc1-3* mutant. (A) Gel image showing splicing variants in the *osabcc1-3* mutant using a cDNA template. (B) Sequence of the PCR product in WT and *osabcc1-3* mutant by Sanger sequencing. Red text indicates the mutation point in the *osabcc1-3* mutant. Gray shade indicates the intron sequence in WT. The underlined sequence indicates a stop codon. Sequencing of Variant 1 failed. (C) Amino acid sequence of (B). * indicates the stop codon. The number after each variant's amino acid sequence indicates the predicted total size (in amino acids) of the protein produced by each variant.

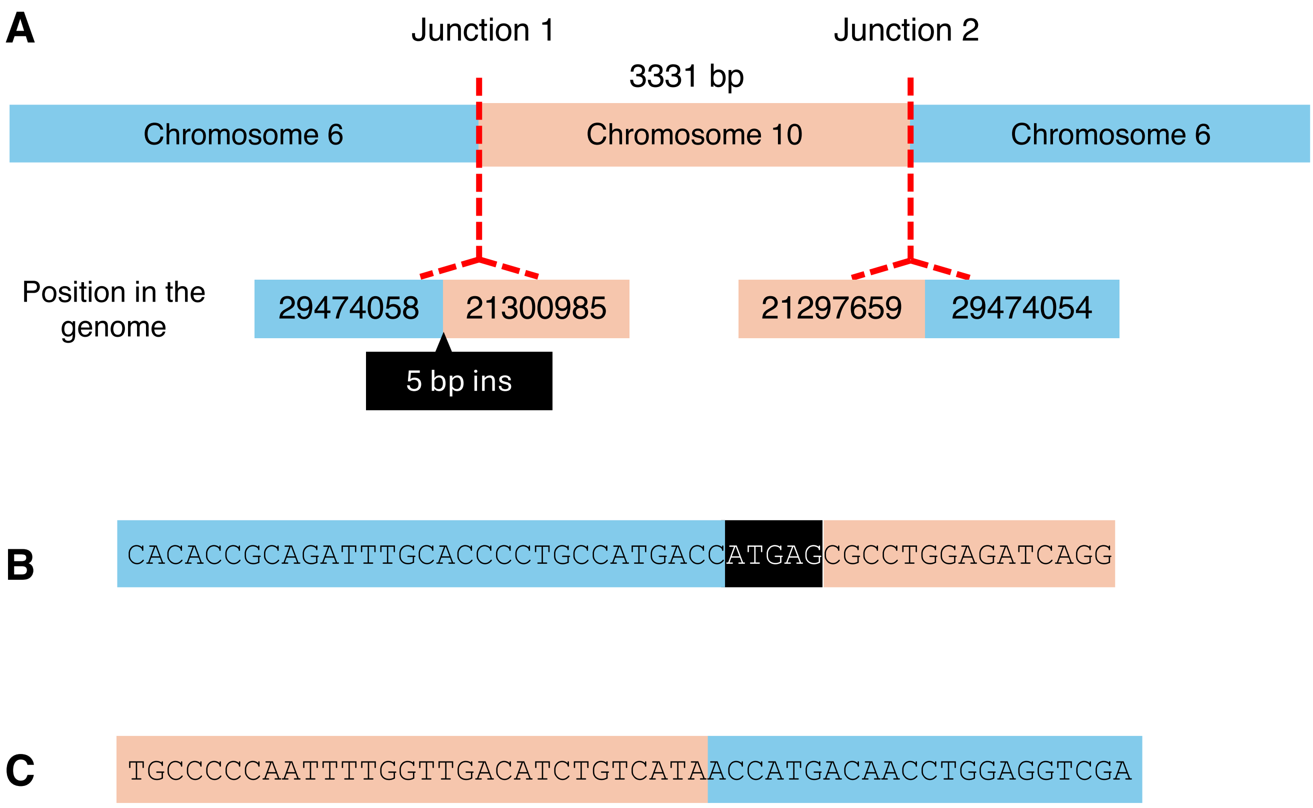

Figure S5 Schematic diagram of the insertion site in the *OsHMA2* gene in the *oshma2-4* mutant. (A) Connection points of the chromosomes 6 and 10 and their positions in the genome. (B) Sequence of the junction 1. (C) Sequence of the junction 2.

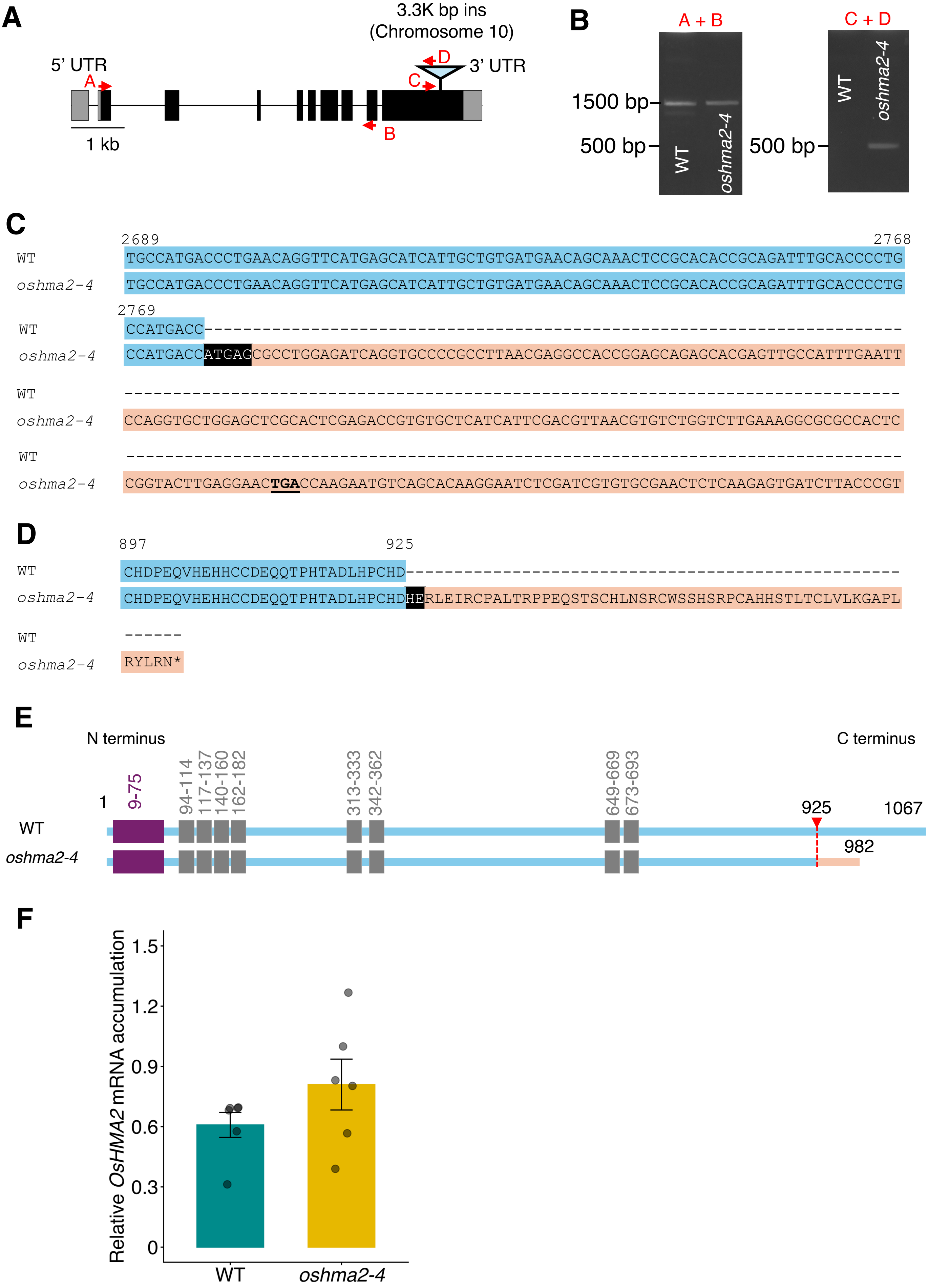
A
3.3K bp ins
(Chromosome 10)
5' UTR
3' UTR
A
B
C
D
1 kb
B
A + B
C + D
1500 bp
500 bp
500 bp
WT
oshma2-4
WT
oshma2-4
C
2689
2768
WT TGCCATGACCCTGAACAGGTTCATGAGCATCATTGCTGTGATGAACAGCAAACTCCGCACACCGCAGATTTGCACCCCTG
oshma2-4 TGCCATGACCCTGAACAGGTTCATGAGCATCATTGCTGTGATGAACAGCAAACTCCGCACACCGCAGATTTGCACCCCTG
2769
WT CCATGACC
oshma2-4 CCATGACCATGAGCGCCTGGAGATCAGGTGCCCCGCCTTAACGAGGCCACCGGAGCAGAGCACGAGTTGCCATTTGAATT
oshma2-4 CCAGGTGCTGGAGCTCGCACTCGAGACCGTGTGCTCATCATTCGACGTTAACGTGTCTGGTCTTGAAAGGCGCGCCACTC
oshma2-4 CGGTACTTGAGGAACTGACCAAGAATGTCAGCACAAGGAATCTCGATCGTGTGCGAACTCTCAAGAGTGATCTTACCCGT
D
897
925
WT CHDPEQVHEHHCCDEQQTPHTADLHPCHD
oshma2-4 CHDPEQVHEHHCCDEQQTPHTADLHPCHDHERLEIRCPALTRPPEQSTSCHLNSRCWSSHSRPCAHHSTLTCLVLKGAPL
oshma2-4 RYLRN*
E
N terminus
C terminus
1
9-75
94-114
117-137
140-160
162-182
313-333
342-362
649-669
673-693
925
1067
982
WT
oshma2-4
F
Relative OsHMA2 mRNA accumulation
1.5
1.2
0.9
0.6
0.3
0
WT
oshma2-4

Figure S6 Effect of insertion in *OsHMA2* transcript. (A) Schematic representation of the positions of primers used for PCR. The arrow indicates the position of each primer. (B) Gel image of PCR products obtained using the primers in (A) with a cDNA as the template. (C) cDNA sequence including junction 1 in WT and *oshma2-4*. Light blue and light peach shading indicate sequences derived from chromosomes 6 and 10, respectively. (D) Predicted amino acid sequence from the sequence shown in (C). * indicates the stop codon. (E) Schematic representation of *OsHMA2* protein in WT and *oshma2-4* mutant based on the UniProt database (https://www.uniprot.org/uniprotkb/A3BF39/entry#sequences). The purple box indicates the HMA domain, the gray boxes indicate transmembrane regions, and the red triangle marks the insertion site in *oshma2-4*. The light peach line indicates the inserted amino acids. (F) Relative *OsHMA2* mRNA accumulation. The mRNA accumulation of *OsHMA2* was determined in the roots of two-week-old plants grown in growth chamber (30°C, 76% relative humidity, 16/8 h light/dark cycle, and photosynthetic photon flux density 690 μmol m^{-2} s^{-1}). The mRNA accumulation level of *OsHMA2* was normalized to the rice *Actin1* gene (mean ± SE; n = 6). The significant differences were calculated using Student's *t*-test ($p < 0.05$).

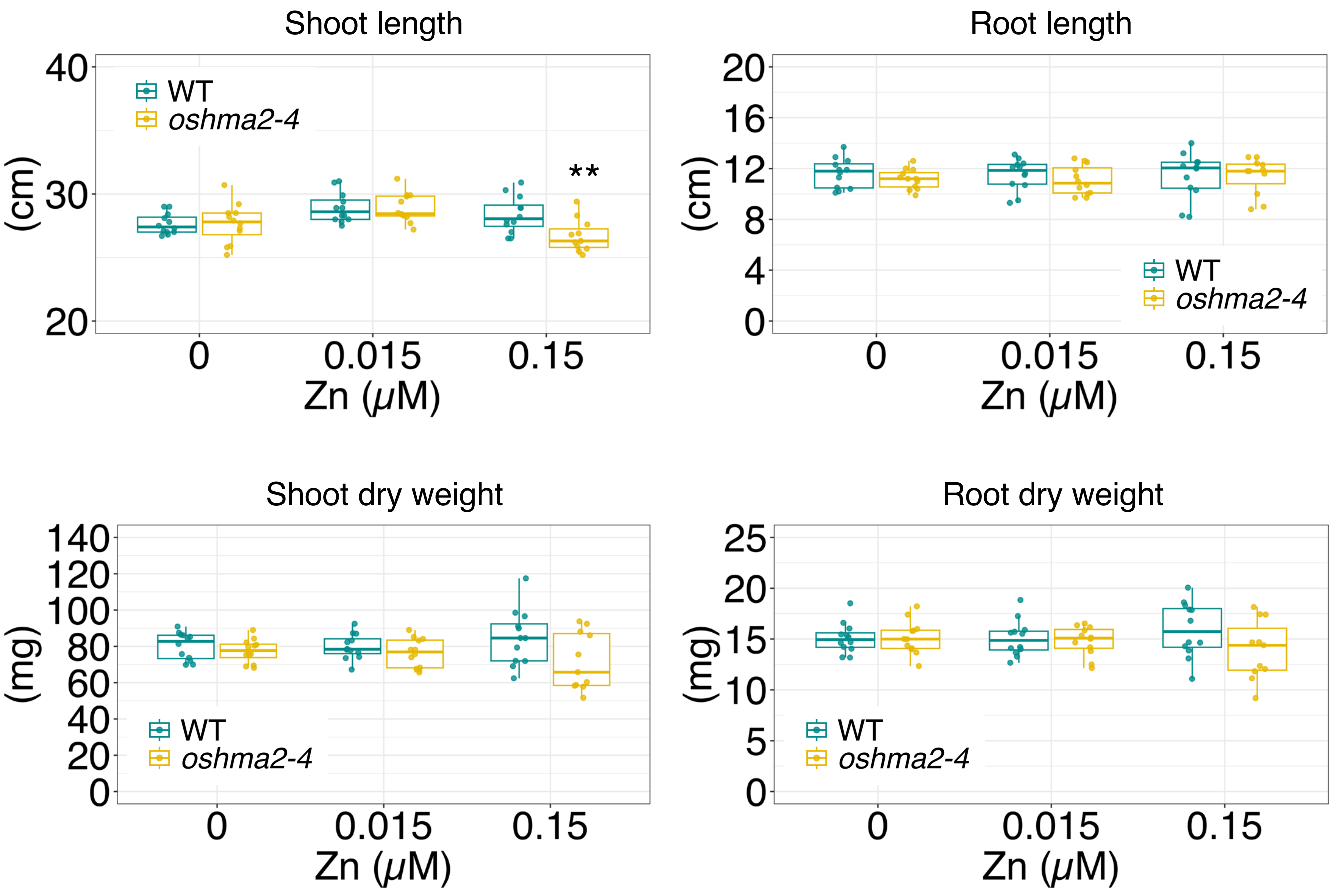

Figure S7 Growth performance of *oshma2-4* mutant under different Zn concentrations in Kimura B for three weeks. n = 11–12, Student's *t*-test, ** $p < 0.01$. Data of Zn (0.15 μM) is the same as in Fig. 7 (Cd 0 μM). The plants were grown in the greenhouse (20–30°C, sunlight with open air).

Table S1 Nutrient composition of the Kimura B solution

Chemical	Concentration
$(NH_4)_2SO_4$	0.7 mM
NaH_2PO_4	0.65 mM
K_2SO4	0.27 mM
$MgSO_4$	0.47 mM
$CaCl_2$	0.37 mM
Fe(III)-EDTA	45 μM
$CuSO_4$	0.16 μM
H_3BO_3	15 μM
Na_2MoO_4	0.1 μM
$MnSO_4$	4.6 μM
**$ZnSO_4$**	**0.15 μM**
MES (pH 5.8)	3 mM

Table S2 The certified values, measured values, and recovery rates of elements in the reference material (SRM 1573a, Tomato Leaves; NIST)

Element	Certified value (ppm)	Measured value (ppm)	Recovery rate (%)
B	33.33 ± 0.42	16.61 ± 1.65	50.1
Na	136.1 ± 3.7	157.88 ± 19.41	116.0
Mg	12000	8427.21 ± 854.98	70.2
P	2161 ± 28	2085.12 ± 138.91	96.5
S	9600	9186.78 ± 419.73	95.7
K	26760 ± 480	20817.27 ± 2155.57	77.8
Ca	50450 ± 550	39724.58 ± 2989.36	78.7
Mn	246.3 ± 7.1	231.56 ± 15.65	94.0
Fe	367.5 ± 4.3	326.63 ± 22.33	88.9
Co	0.5773 ± 0.0071	0.44 ± 0.03	76.7
Ni	1.582 ± 0.041	1.15 ± 0.10	72.9
Cu	4.7 ± 0.14	5.13 ± 0.40	109.2
Zn	30.94 ± 0.55	29.13 ± 1.90	94.1
As	0.1126 ± 0.0024	0.12 ± 0.01	107.7
Sr	85	70.73 ± 5.54	83.2
Mo	0.46	0.31 ± 0.03	67.0
Cd	1.517 ± 0.027	1.30 ± 0.09	85.6
Cs	0.053	0.032 ± 0.003	60.2

Table S3 Primers used in this study

Primer name	Sequence	Chr. No.	Purpose
*OsHMA2*_F	AGGCAGCTTCACTATGCTGT	6	To amplify region including the mutation site
*OsHMA2*_R	CCAATGACCATGCTTGCCAT	6	
*OsHMA2*_Junc1_F (C)	GCCATGACCATGAACAGGTT	6	To determine junction 1 sequence
*OsHMA2*_Junc1_R (D)	CCATGCCCCTTAGTGCAATT	10	
*OsHMA2*_Junc1_Seq	AGACAGTCAAACAGGGATGA	10	
*OsHMA2*_Junc2_F	AGCTTCACTATGCTGTGGCT	6	To determine junction 2 sequence
*OsHMA2*_Junc2_R	CGCACCAAACTAGCAACAGA	10	
*OsHMA2*_Junc2_Seq	GCAAGCTCGAGCAAATGTCT	6	
*OsHMA2*_Exon2_F (A)	AGAAGAGCTACTTCGACGTG	6	To amplify the regions from exon 2 to exon 9
*OsHMA2*_Exon9_R (B)	CCTCAGCTGATCCAGTTCGG	6	
*OsHMA2*_qPCR_F	AGAAGAGCTACTTCGACGTG	6	To determine mRNA accumulation by qPCR
*OsHMA2*_qPCR_R	GATTCAGCGCCTTGACGATT	6	
*Actin1*_qPCR_F	CTGCGGGTATCCATGAGACT	3	
*Actin1*_qPCR_R	TGGAATGTGCTGAGAGATGC	3	
*OsABCC1*_dCAPs_EcoR V_F	GAAAATATAGCTATCATCTGTACA AAGATA	4	To determine the genotype of F2 population of WT × *osabcc1-3*
*OsABCC1*_dCAPs_EcoR V_R	CCTTATAATCTTTCCTCAGGTTCC	4	
*OsABCC1*_cDNA_F	ACCAAAGCCTTGGCTGTTAC	4	To check the splicing error in *osabcc1-3* mutant
*OsABCC1*_cDNA_R	AGTGACACTCCAGCGAAGAT	4	
*OsABCC1*_cDNA_Splicing_F	TGTTACGGGCTCTGCATAGC	4	To determine the sequence of each splicing variants by Sanger sequencing

Note:

- *OsHMA2*_F and *OsHMA2*_R produced a 650 bp PCR product in WT and a 4 kbp PCR product in *oshma2-4*. Again, *OsHMA2*_Junc2_F and *OsHMA2*_Junc2_R produced a 500 bp PCR product in *oshma2-4*. These primer sets were used for genotyping of F2 population of *1%EMS_L13* × *oshma2-4*.
- *OsABCC1*_dCAPs_EcoR V_F and *OsABCC1*_dCAPs_EcoR V_R produced a 130 bp PCR product in both WT and *osabcc1-3* mutant, and after digestion with EcoR V enzyme, the band size was 130 and 100 in the WT and *osabcc1-3* mutant, respectively.
- A, B, C, and D primers are used in **Fig. S6A**.
- *OsHMA2*_Exon2_F and *OsHMA2*_qPCR_F are same.
